# Supplementary material for: Are carbon emissions trading and green financial instruments synergistic? -Comprehensive quantitative research based on content analysis
Source: PLoS One. 2024 Mar 7;19(3):e0298601. doi: 10.1371/journal.pone.0298601 (PMC10919601; doi:10.1371/journal.pone.0298601)
Supplement: S1 Data — (ZIP) [file pone.0298601.s001.zip › Data/地级市Z_F工作报告.zip!/╣π╬≈/║╪╓▌╩╨/└┤╫╘╣1⁄2╓┌║┼í╛┬φ┐╦╩2╛▌═°í┐_2013.docx]

各位代表:

　　现在,我代表市人民政府向大会作工作报告,请予审议,并请市政协委员和其他列席人员提出意见。

2012年工作回顾

　　2012年,面对复杂多变的经济形势,全市上下深入贯彻落实科学发展观,按照市委提出的“稳中求进、进中求快、快中保好”的主基调,着力转方式、调结构、扩内需、惠民生、促和谐,基本完成了市人大三届二次会议确定的目标任务,保持了经济社会平稳较快发展势头。

　　一、经济建设迈上新台阶。全年有12项主要经济指标保持两位数增长,有6项主要经济指标实现在全区排位前移。据统计,全市实现地区生产总值393.9亿元,增长9%；财政收入32亿元,增长20.2%,增速排全区第3位；全社会固定资产投资591.6亿元,增长26.9%,连续四年保持每年100亿元增量；规模以上工业总产值301亿元,增长20.7%,增速排全区第6位；规模以上工业增加值95.1亿元,增长12.3%；农业总产值135.6亿元,增长6.1%；社会消费品零售总额106.4亿元,增长15.2%；城镇居民人均可支配收入19855元,增长12.8%,增速排全区第5位；农民人均纯收入5823元,增长17.3%,增速排全区第3位,城乡居民收入增速均高于地区生产总值增速。非公有制经济稳步发展,实现增加值310亿元,占全市经济总量的78.7%。

　　——工业经济持续增长。全部工业总产值突破400亿元,达到411.3亿元,增长16.1%,荣获自治区工业产业发展优秀奖。启动稀土、钨钛锡、碳酸钙新材料产业园建设,碳酸钙新材料产业示范基地列入自治区重点建设基地,碳酸钙产业成为我市第二个产值超50亿元产业。加快发展重点园区,六大产业园区实现工业总产值245亿元,占全部工业总产值的59.6%。大力实施“抓大壮小扶微”工程,净增规模以上工业企业17家,新增亿元企业17家,新增微型企业631户。突出抓好重大产业项目建设,完成工业投资305.3亿元,增长39.2%；完成技改投资145.3亿元,增长45.3%。

　　——现代农业加快发展。创建广西出口食品农产品质量安全示范区实现全覆盖,出口备案基地达到21个,面积增至18.3万亩,八步区成为国家蔬菜清洁生产示范项目县。粮食生产实现“九连增”,总产量增产1.98万吨,达到71.9万吨。建立无公害标准化规模养殖示范场149个,其中自治区级标准化规模养殖示范场3个；市级以上重点农业产业化龙头企业达45家。提升农业科技推广应用能力,开展农民实用技术培训15万人次,主导农作物良种覆盖率达到95%以上。成功探索“富川土地流转新模式”,富川瑶族自治县被农业部列为广西首个“全国农村土地承包经营权流转规范化管理和服务试点地区”项目县。

　　——商贸旅游业发展壮大。各类市场主体较快增长,新增私营企业1487户、农民专业合作社90户、个体工商户6598户。城乡消费市场保持活跃,商品房销售面积增长21.8%,家电下乡产品销售额增长58.8%。金融业服务实体经济的能力增强,金融机构存款余额348.8亿元,增长16.7%；各项贷款余额204.7亿元,增长18.8%,增速排全区第3位,其中小微企业贷款达到62亿元,增长62.8%。新增旅游星级饭店3家,新增国家2A级旅游景区2个,黄姚古镇旅游区荣获“广西五星级乡村旅游区”称号。全年接待游客总人数811.9万人次,增长22.5%；实现旅游总收入72.6亿元,增长32.6%；旅游创汇7630万美元,增长20.7%。荣获“2012中国最佳生态旅游示范城市”称号。

　　——项目建设掀起高潮。继续开展“项目建设年”活动,全年在建施工项目3441项,增长37.2%；新开工2917项,增长46.1%；竣工2682项,增长42.7%。组织实施自治区层面项目22项,完成投资46亿元；市级层面项目159项,完成投资143亿元。争取到国家和自治区投资项目368项,获得资金13.5亿元,增长39.2%。交通、水利电力等基础设施得到有效改善,完成交通运输固定资产投资54.6亿元,黄姚至昭平、白沙井至西坪二级公路建成通车,望高至富川二级公路改造项目竣工,农村客运站覆盖率居全区前列；完成水利电力固定资产投资37.1亿元,江河治理、饮水安全保障、病险水库除险加固和农田水利　“四大工程”　建设加快推进。

　　——城镇化建设成果丰硕。围绕开展建市十周年系列纪念活动和“五城联创”活动,建立了城建重点项目快速推进非常机制,加快实施城市基础设施建设大会战。21个重点城建项目完成投资19.12亿元,其中市传媒中心、灵峰大桥、贺州至姑婆山道路二期工程等12个项目已完工或基本完工,绿洲家园、太白湖公园、市文化中心等9个项目加快推进。城市建成区人口新增1.7万人,达到30万人；面积增加2平方公里,达到34平方公里。再获“全国双拥模范城”称号,荣获全区第八届市容“南珠杯”优秀城市奖、“广西森林城市”和“广西卫生城市”称号,“国家森林城市”创建工作进展顺利。实现中心城区控制性详细规划和乡镇总体规划全覆盖,城乡风貌改造四期工程开竣工率排全区前列,深入推进特色名镇名村和新农村示范点建设。全市城镇化率提高2个百分点,达到39%。

　　——县域经济竞相发展。出台实施《关于加快县域经济发展的实施意见》及配套文件,严格实行发展任务和发展成效“双考核”。推进扩权强县改革,下放行政审批项目558项。各县(区、管理区)根据区域特色,突出优势、奋勇争先,县域经济加快由农业支撑向工业主导转变,地区生产总值超50亿元的县(区、管理区)达到4个。

　　——改革开放步伐加快。有序推进第三批事业单位实施绩效管理工作。积极促进平桂管理区加快资源枯竭型城市转型发展。完成市县文艺院团改革主体任务和市新华书店改制。新设立首家村镇银行——八步东盈银行、首家农村商业银行——昭平农商行。加强与梧州、玉林、贵港三市务实合作,加快打造国家级桂东承接产业转移示范区。主动与广东佛山、肇庆对接,共同推进粤桂产业合作八步—怀集示范区建设。深化与华润集团、中国铝业、中国有色等央企合作,加快推进“央企入贺”合作项目建设。全市投资规模50万元以上的招商引资项目到位资金347.2亿元,其中外资项目到位资金9000万美元；实现外贸进出口总额1.56亿美元。

　　二、生态建设开创新局面。全面实施“十二五”节能减排综合性工作方案,30个减排项目全面完成,8家自治区重点监控用能企业累计节能超万吨标准煤。对辖区内涉及重金属排放的采选、冶炼等140多家企业排查整治、倒逼升级,五大钢铁企业实现产值占全部工业总产值的比重下降1个百分点。大力发展循环经济,贺州华润循环经济产业示范区被评为省级循环经济园区和自治区A类产业园区,华润(贺州)电厂2台机组建成运行,华润雪花啤酒建成投产；积极推广以沼气为纽带的生态循环农业模式,农村沼气池入户率继续居全区首位。继续实施贺江和城市空气“清新行动”、“绿满贺州”造林工程,森林覆盖率达72.16%。

　　三、文化建设呈现新气象。邀请中央电视台“心连心”艺术团来贺州演出并获成功,创作大型舞剧《瑶妃》并荣获桂花金奖,推出贺州原创歌曲专辑并成功拍摄《莲花》MTV播出,成功举办全国书法小品大赛、“大美贺州”玉石展,丰富了建市十周年纪念活动内容。“国学进万家”活动深入人心,“千村万户文艺惠民工程”顺利实施。征集提炼“重诚厚德、开放包容、自强不息”的贺州精神,评选莲花为市花、樟树为市树。全面完成80个村级公共服务中心建设。

　　四、社会建设取得新进步。加快以民生为重点的社会建设,民生领域财政支出达77.5亿元,增长24.2%,占公共财政预算支出的79.3%。13项为民办实事工程基本完成。深入实施第五轮创新计划,专利申请量增长30.8%,专利授权量增长16.3%。深入实施中心城区教育项目建设大会战,贺州高中迁建、贺州二高扩建等工程顺利推进,荣获“全区职业教育攻坚工作进步市”和“全区职教攻坚工作职教园区建设创新市”称号。新增城镇就业1.57万人,新增农村劳动力转移就业3.55万人,城镇登记失业率为3.28%。实现城乡养老保险制度全覆盖,基本养老、基本医疗、失业、工伤、生育保险参保人数累计达59.38万人次；新型农村合作医疗参合率达到97.12%。钟山县率先在全区建设县级社会福利及居家养老休闲中心,被民政部列为民政综合改革和理论创新基地。居民消费价格指数涨幅2.8%,排全区第11位。人口自然增长率控制在7.92‰。实施新一轮扶贫开发攻坚战,启动钟山县等革命老区扶贫攻坚三年帮扶行动。解决14.5万农村居民饮水安全问题。新开工保障性住房9020套,竣工5600套。消除农村茅草树皮房693户,完成农村危房改造1.64万户。全面完成年度医改任务,医药卫生服务体系不断完善,市人民医院医技综合楼即将投入使用,在全区率先创建“全国白内障无障碍市”。成功举办市第三届运动会暨首届全民健身运动会。国防教育和国防后备力量建设深入开展。安全生产工作完成自治区下达事故控制考核年度指标,再次被评为优秀等次。无重大食品药品安全事件发生。实现十八大期间赴邕进京“零上访”目标,市城区实现累计187天“两抢”案件零报警,农村社会治安管理新模式得到自治区高度评价,社会公众安全感明显提高。

　　五、政府自身建设有新成效。以开展“解放思想、赶超跨越”大讨论活动为契机,创新工作机制体制,出台实施一系列保农业、促工业、抓投资、稳增长的政策措施,破解了诸多发展“两难”问题。深入开展“治庸、治懒、治散”活动,探索推行“一线工作法”,工作作风更加务实。坚持政府常务会学法制度,健全完善决策规则程序,依法行政水平有新提高。重大事项及时向市委报告并呈市委审定,自觉接受市人大及其常委会的法律监督、工作监督和市政协的民主监督。办结各类网上审批事项12.5万件,办理审批提速97.56%。45个乡镇政务中心实现挂牌办公,拓宽了与群众沟通联系的渠道。积极开展保持党的纯洁性“以案明纪”警示教育活动,不断加强行政监察和审计监督工作,立案查办各类违纪违法案件126件,核查处置违规公务用车778辆,“三公”经费支出控制在预算规模内。

　　我们还加强了与各民主党派、工商联、无党派人士和工会、共青团、妇联的联系,统计、烟草、邮政、通信、气象、水文、老龄、残联、档案、地方志、机构编制、应急管理、行政服务、决策咨询、驻梧管理、住房公积金、质量监督、检验检疫、防震减灾、库区移民、外事侨务、民族宗教等工作也取得了新成效。

　　各位代表,这些成绩是在经济下行的巨大压力下取得的,来之不易,是全市人民共同努力的结果,也是社会各界关心支持的结果。在此,我代表市人民政府,向辛勤工作在各条战线的广大干部群众,向给予政府工作大力支持的人大代表和政协委员,向驻贺部队、武警官兵和政法干警,向各民主党派、工商联、人民团体和社会各界人士,表示衷心的感谢并致以崇高的敬意!

　　在看到成绩的同时,我们也清醒认识到发展中还存在不少困难和问题,主要是:受宏观政策和市场需求影响,部分主要经济指标增速低于预期发展目标；县域经济发展不平衡；项目建设要素瓶颈制约严重,资金保障和土地承载明显滞后于项目建设需求；保工资、保民生、保项目建设配套资金等刚性支出剧增,财政收支矛盾突出；群众对美好生活的向往更加强烈,就业、上学、医疗、养老、安居等民生需求更高；社会管理领域还存在一些薄弱环节；环境保护工作任重道远；新形势下群众对政府工作有更高要求,政府加强自身建设和推进改革发展的任务更加繁重。对此,我们要运用“底线思维”的方法要求,努力把2013年工作做好。

2013年工作展望

　　根据中央、自治区的要求和市委的部署,2013年政府工作的指导思想是:深入学习和全面贯彻落实党的十八大精神,大力实施“工业立贺、富民强市”战略,以全力推动工业化、城镇化和农业现代化为重点,加大经济结构调整力度,提高经济增长质量和效益,实现财政增长、城乡居民收入增加目标,深化改革开放,推动文化繁荣发展,打造特色宜居城市,建设美丽贺州,切实保障改善民生,促进各项事业协调发展,为力争与全国全区同步全面建成小康社会打下坚实基础。

　　全年经济社会发展的主要预期目标是:地区生产总值增长11%；财政收入增长12.5%；全社会固定资产投资增长15%；规模以上工业增加值增长18%；农业总产值增长6.5%；社会消费品零售总额增长16%；外贸进出口总额增长13%；直接利用外资增长10%；万元生产总值能耗下降2.8%,二氧化硫、氮氧化物、化学需氧量和氨氮排放总量分别控制在7089吨、10752吨、9920吨和470吨以内；城镇居民人均可支配收入增长12%；农民人均纯收入增长13%；居民消费价格指数涨幅控制在4%左右,城镇登记失业率控制在4.5%以内,人口自然增长率控制在9‰以内。

　　要实现今年的工作目标,必须做好应对更大困难和挑战的准备。当前,受世界经济复苏乏力、国内外市场需求萎缩、经济下行压力加大等因素影响,我市经济企稳回升势头缓慢。国家经济转型发展倒逼升级力度加大,贺州以资源型为主的工业产业结构调整步伐亟需加快。随着桂梧高速公路、广贺高速、洛湛铁路贺州段等重点项目陆续完工,贵广快速铁路贺州段等项目加快推进,后续重点项目缺乏,投资增长支撑不足。中央坚持房地产调控政策不动摇,我市冶金、建材等重点税源企业生产将继续受到较大影响,而且受国家结构性减税、税收优惠政策和地方政府债务进入偿债高峰期等因素影响,我市将面临巨大的财政压力。中央继续实施稳健的货币政策,扩大直接融资规模难度很大,建设资金瓶颈问题短时间内难以缓解；全市节能减排基数小,华润(贺州)电厂等一批高耗能企业投产运营后,节能降耗工作压力极大；收储土地不多,征地拆迁难度大,土地供应难以满足项目建设需要,这些刚性约束极大地制约着贺州后发优势的发挥。

　　当然,我们也面临着许多难得的发展机遇。世界经济缓慢回升,东亚地区成为推动全球经济增长的重要一极,为我市在原来基础上扩大外贸进出口规模创造了有利条件。我国经济发展的基本面趋好,中央继续实施积极的财政政策,积极稳妥推进城镇化,有利于我们加快改善发展中的薄弱环节。贺州资源优势突出,大理石、钨、锡、稀土等储量位居广西前列,建成或在建水电、火电、风电、太阳能等装机容量达到300多万千瓦,农林牧渔资源丰富,转化为经济优势的潜力巨大。“五高三铁两江一机场”立体交通格局加快构建,市内交通条件不断改善,正逐渐形成市域1小时交通圈和融入珠三角2小时经济圈、北部湾3小时经济圈。国家级桂东承接产业转移示范区、粤桂产业合作八步—怀集示范区加快建设,产业合作平台日益完善,开辟了承接东部产业转移的广阔空间。同时,我市正处于迈向工业化、城镇化中期阶段的快速发展期,城市发展格局正发生新变化,电力、林产、矿业、电子和新材料等支柱产业初具规模,一批竣工企业陆续达产,将进一步提升我市综合竞争实力。

　　困难虽是挑战,机遇蕴含希望,办法来自决心。只要我们统一思想,坚定信心,牢牢把握主动权,奋发有为地做好各项工作,就一定能化挑战为机遇,变压力为动力,保持全市经济社会持续健康发展。

　　我们要重点做好以下方面工作:

　　一、加快推进农业现代化,多渠道增加农民收入

　　深入实施万元增收工程、“234”农业产业化工程和三大增粮增收行动,继续把农民人均纯收入目标列入党政领导班子的重要考核内容,确保全市农民人均纯收入达到6400元以上。

　　加快发展现代农业,努力提高农民家庭经营性收入。大力挖掘农业内部增收潜力,优先发展优质粮,确保粮食播种面积稳定在208万亩以上、总产量70万吨以上；加快发展昭平茶叶、富川脐橙、钟山贡柑、平桂马蹄、八步蔬菜等优势特色种植业；大力发展林下经济,提升森林经营质量和效益；因地制宜抓好生猪、优质家禽和名优水产品等优势特色养殖业；抓住自驾游、自助游需求不断增长的有利时机,发挥农业的观光旅游、休闲度假功能,积极发展乡村旅游。

　　提高农业产业化水平,有效增加农民工资性收入。抓好贺州温氏公司等重点农业产业化龙头企业生产服务工作。充分发挥龙头企业的辐射带动作用,鼓励农民兴办各类新型合作组织,密切龙头企业与基地、农户的产销联系,从产供销各个环节增加农民收入。加强职业技能培训,开展区域劳务合作,鼓励和帮扶外出务工农民回乡创业,加大农村劳动力转移就业力度。

　　落实强农惠农富农政策,不断提高农民转移性收入。严格落实财政支出重点向农业倾斜政策和各项补贴政策；落实新农合基金补助政策,对新农合补助标准提高到年人均280元,确保新农合参保率达到95%以上；完善农村“一事一议”筹资筹劳办法；按计划完成农村危房改造任务。

　　建立完善产权制度,让农民获得更多财产性收入。加强农村资金、资产、资源规范化管理。完善并推广“富川土地流转新模式”,按照依法自愿原则,鼓励和支持承包土地通过转包、互换、转让、股份合作等形式,向专业大户、家庭农场、农民合作社流转,扩大农村土地、林权等收益。

　　积极创建农业标准化示范市,以农产品质量提升促进农民增收。充分发挥贺州在广西率先实现出口食品农产品质量安全示范区全覆盖的优势,新建出口备案基地5个,新增面积5万亩,新增规模农业企业5家。启动农业标准化示范市创建工作,增强全市农业主导产业发展能力,提高名优农产品在粤港澳地区的市场份额。

　　健全农业社会化服务体系,增强农民增收保障能力。积极探索进城就业、创业、务工农民社会化管理服务模式。鼓励和引导金融机构加大对农村地区信贷投入和保险支持力度,保持小微企业和涉农贷款不低于全部贷款增速。推动现代流通方式向农村延伸,推进金泰农产品加工物流中心、富川特色农产品加工物流中心等建设；深化“万村千乡市场工程”和农贸市场升级改造；抓好“农超对接”试点工作。加强高标准基本农田水利建设,加快推进病险水库除险加固、中小河流治理、大中型灌区配套设施与节水改造、小型农田水利建设。

　　二、落实政策鼓励创业,稳步提高城镇居民收入

　　贯彻落实积极的就业政策,强化政策扶持、创业培训、创业服务“三位一体”工作机制,实施全民创业工程,确保城镇居民人均可支配收入达到21900元以上。

　　推动实现更高质量的就业。加快发展商贸流通、家政、养老、社区服务等生活性服务业,积极发展信息咨询、电子商务、科技服务等生产性服务业,多渠道开发就业岗位,促进城镇居民增收。重点做好高校毕业生就业服务工作,加强对城镇低保对象、困难职工的就业援助,实现城镇新增就业1.6万人。

　　积极促进全民创业。全面落实国家、自治区和我市鼓励民间投资和民营经济发展的政策措施,完善面向民营企业的投资融资、技术支持、创业服务等公共服务平台。继续实施微型企业创业扶持管理办法,力争新增微型企业1000家以上,安排就业人员8000人以上。

　　完善企事业收入增长机制。健全企业工资分配机制,推进企业工资集体协商制度和最低工资制度。加强劳动者工资水平与企业效益有机衔接。建立预防和解决工资拖欠的长效机制,增加低收入群体收入。积极推进事业单位收入分配制度改革,加快推进事业单位实施绩效工资。

　　提高城镇社会保障统筹层次和保障水平。强化失业、工伤、生育等社会保险扩面征缴。根据我市经济社会发展水平和财力状况,建立健全社会保险待遇正常增长机制。进一步完善各项社会救助制度,为各类特殊困难群体提供社会救助。

　　三、加快推进新型工业化,推动产业转型升级

　　全方位服务企业发展。对全市规模以上工业企业采取“一企一策”办法,协助企业解决生产经营管理难题,提高煤电油运资等生产要素保障能力,特别是积极争取企业技改资金、特殊行业生产指标、产业用地指标、工业企业分时段计价用电等倾斜政策,支持企业创新发展,促进停产企业恢复产能、新建成或技改竣工企业达产,夯实经济增长基础。

　　加快构建“6+3”现代产业体系。尽快把电力、建材、造纸与木材加工、冶金、电子信息和新材料产业打造成六大百亿元产业,同时优化提升发展食品、有色金属、机械制造等三大产业集群发展。开工建设桂东电子四期技改、扬翔饲料等项目,加快推进平桂飞碟钨系列产品深加工技改、桂海铝业电子铝光箔等项目建设。

　　加大工业结构调整力度。通过实施技术更新改造,推动水泥、碳酸钙、有色金属等传统优势产业调整升级,重点推进碳酸钙新材料产业示范基地建设。力争完成技改投资174亿元以上。运用环境倒逼机制淘汰落后小企业,大力发展战略性新兴产业,加快推动以稀土、黑钨为主导的新材料产业。深入实施“质量兴贺”战略,加强质检中心建设,开展贺州市政府质量奖评审活动。

　　加快工业园区扩容提质步伐。重点推进贺州华润循环经济产业示范区污水管网一期工程、石材循环经济产业园区路网一期工程等园区基础设施项目建设,完善六大产业园区基础功能和公共服务体系。完善以信都至富川为主干的高效路网,加快构建百里工业长廊,提升产业发展承载能力。

　　深入实施“抓大壮小扶微”工程。力争亿元企业达到68家以上,形成梯次强企方阵。鼓励主营业务收入2000万元以下企业,通过增加投入、盘活存量、兼并重组等方式做大规模,力争规模以上企业总量达到175家以上。通过总量扩张和转型升级,力争实现工业总产值500亿元,工业增加值164亿元,其中规模以上工业总产值360亿元,规模以上工业增加值116亿元。

　　推进工业化与信息化深度融合。深入推进“无线城市”、“数字贺州”建设,推动电信、数字电视、互联网“三网融合”。着力加强工业经济系统网络建设,构建满足管理部门和中小企业共性需求的公共信息平台。引导企业信息化改造,鼓励企业开展信息化应用,积极探索以信息化促进稀土、电子等新材料产业创新升级新路径。

　　四、加快推进新型城镇化,打造特色宜居城市

　　加强城乡规划管理。依托优美的山水风光,发掘地方文化特色,用先进的理念完善城市规划,优化城镇空间布局,搞好新农村建设规划,形成山水宜居、各具特色的城乡风貌。重视把城镇化和调整产业结构、培育新兴产业、发展服务、促进就业创业结合起来,推动城镇化工业化互动发展。巩固扩大“五城联创”创建成果,拓展延伸城乡清洁工程,着力改善旧城区、城乡结合部、乡镇卫生环境。完善市容环境综合整治长效机制,加强道路通行秩序管理,整治违法乱建、乱摆乱卖等突出问题,努力改善百姓的生活环境。推进黄姚、莲山等重点建制镇污水垃圾处理设施建设,探索实施有效的农村生活垃圾处理方式。

　　加大城区建设力度。加快推进以城东新区、平桂新区为重点的城市新区建设,重点推进贺州大酒店、绿洲家园、太白湖公园、城东新区路网、平桂新城路网工程等城建项目。抓好城市基础设施建设,开工建设城区排水排污及供水管网改造、担杆岭自来水厂二期扩建工程、平桂污水处理厂等项目。积极谋划新的城区建设,将周边的黄田、沙田、鹅塘、莲塘、贺街逐步纳入城区建设管理,拉大城市框架,尽快启动北环路、灵峰南路、莲塘城北大道等建设,构筑便捷高效的城市主干路网,加快形成“一江两岸三新区”的城市发展格局。

　　加快建设现代城镇体系。以“贺八平钟一体化”为目标,积极构建以市区为中心,钟山为副中心,富川、昭平县城为卫星城的城镇整体布局,力争全市城镇化率达到41%。加快县城和中心镇建设,着力推进各地新城区、百里工业走廊中心城镇和重点镇的基础设施、公共设施建设,继续推进城乡风貌改造、名镇名村和新农村示范点建设,促进城镇基础设施向周边农村延伸。

　　提速发展县域经济。努力朝着将八步区、平桂管理区、钟山县建设成为全区县域经济强县,将昭平县、富川瑶族自治县建设成为全区县域经济发展特色县的目标奋进,力争各县(区、管理区)经济增速都达到10%以上,地区生产总值全部超过50亿元,其中超100亿元县区达到2个；二三产业比重分别提高1.5个百分点以上；规模以上工业增加值占全部工业增加值比重达到70%以上。

　　五、发挥投资的关键作用和消费的基础作用,拉动经济稳步增长

　　统筹推进重大项目建设。启动“十大工程”三年(2013-2015)行动计划,谋划推进总投资648亿元的100项重点项目,为调结构、促转型、增实力、保民生提供重要保障。计划实施2013年市级层面项目205个,年度计划投资177.2亿元,重点推进81个亿元以上续建项目建设,力争完成投资95亿元；开工建设26个工业项目建设,力争完成投资20.2亿元；扎实做好一批重大项目前期工作,力争取得实质性进展。通过狠抓重点项目建设,确保全社会固定资产投资完成670亿元。

　　加强交通和能源、水利等基础设施建设。沟通促进广贺高速公路怀集段尽快建成通车,协调推进贵广快速铁路贺州段、永贺高速公路贺州段项目建设,着力推进柳韶铁路贺州段、韶百高速公路贺州段、洛湛铁路复线及其电气化改造工程等项目前期工作。实施国道323平桂至钟山提级工程、昭平绕城线等路网工程和一批农村公路建设。加快建设上程电站、路花水库水源、贺州主电网、城区电网、农村电网升级改造等工程。

　　积极破解项目建设瓶颈。促进金融机构加大对符合条件的产业项目、民生项目和重大基础设施项目建设资金的投放力度,力争金融机构存款余额新增55亿元以上,增长15%以上；各项贷款余额新增35亿元以上,增长16.7%以上。突出抓好征地拆迁工作,确保重点项目建设顺利推进；推进城乡建设用地增减挂钩,强化土地节约集约利用；加大闲置土地处置力度,整合盘活土地资源；做好城东新区、江南新区、平桂新区和各重点产业园区的土地收储工作,合理开发利用城市建设用地；统筹落实用地指标700公顷以上,保障项目建设用地。

　　继续强化项目管理。坚持市四家班子联系重点项目建设制度,进一步创新重大项目推进机制,完善“六个一”等行之有效的项目推进模式。继续简化项目审批手续,压缩审批时限,实现项目审批各个环节全面提速。执行更加严格的绩效考核和奖惩问责制度,及时对市级层面重大项目建设工作进行督查和考评奖罚。

　　加快发展非公有制经济。不断优化投资环境,继续推行市领导联系非公有制企业制度,探索为非公企业办理行政审批手续提供“一站式”、“点对点”服务。最大限度取消民间资本投资限制,鼓励民间资本进入市政设施、医疗事业、教育事业、文化产业等领域。鼓励引导民间资本进行重组联合、参与国有企业改革。力争年内新增私营企业1500户,农民专业合作社90户,个体工商户7000户。

　　发展壮大旅游业。实施“富民兴旅”三年行动计划。推进贺州市一景地质矿产资源旅游景区开发、大桂山生态旅游区开发、钟山县十里画廊慢城度假旅游开发等项目建设,推进姑婆山国家森林公园、黄姚古镇景区创建国家5A景区工作。加快融入大桂林旅游圈,打造华南五市山水休闲旅游黄金专线品牌,拓宽旅游客源市场,确保实现接待游客总人数增长20%、旅游总收入增长30%。

　　努力改善消费需求结构。积极发展新型消费和绿色消费,丰富消费产品类型,培育新的消费热点。落实中央鼓励消费的各项政策,推动批发、零售、住宿、餐饮等行业加快发展。提升贺州农产品展销会、房车展销会等会展经济影响力。加快智能交通、安防监控、数字社区等物联网重大应用。着力构建多层次保障性住房体系,年内完成5688套保障性安居工程目标任务。有序推进敬老院等养老服务设施建设。加大中小户型、中低价位普通商品住房建设力度。坚决打击商业欺诈、制假售假行为,让广大群众方便、放心、安全消费。确保全社会消费品零售总额达到124亿元以上。

　　六、加强财政收支管理,确保实现财政增长目标

　　加强税源培植。发挥税收的经济杠杆作用,综合运用贴息、补助等政策手段,加大对重点企业的扶持力度,提供新的税源增量支撑。加强在建项目的跟踪服务,力促一批重大项目竣工投产,尽快产生效益。抓好重大项目引进,不断发展后续财源。搞好国有资产经营和管理,盘活闲置资产,构建多元化的财源增长体系。

　　强化收入管理。将除教育收费以外的各项非税收入全部纳入预算管理,密切财政、税务、建设等相关部门协作,采取先税后证、以票管税、源泉控管、以电控税等综合措施,重点抓好餐饮业、石材加工企业等专业化税收征管,确保完成财政收入36亿元以上。

　　科学安排支出。强化财政绩效管理和政府债务管理,推进国库集中支付、公务卡制度和政府采购监督管理。做好转移支付、专项补助、债券转贷等新增资金安排。在保工资、保稳定、保民生、保法定支出、保还债的基础上,多挤出财力支持项目建设、生态建设和社会管理创新。

　　七、不断深化改革开放,增强发展动力活力

　　深化重点领域和关键环节的改革。积极探索更加高效的行政管理体制机制,市本级侧重利用产业园区平台,整合各种资源,推动优质企业、项目入园,促进工业发展；各县(区、管理区)在市里的统一指导下,侧重加快推进城镇化、农业现代化步伐。推进重点产业园区管理体制改革,建立科学考核指标体系,完善和理顺各县(区、管理区)与辖区内产业园区之间的权责利关系,推动园区加快发展。加强国有资产监督管理,深化国有企业改革,重点推动贺达纸业改革重组。深化投融资体制改革,增强政府投资的引导和放大作用,引导重点企业有步骤、分重点推进上市工作。深化科技体制改革,加快创新体系建设。稳妥推进事业单位分类改革。

　　加强多层次宽领域的开放合作。抓住粤桂、桂港合作重大机遇,深入实施“东靠”战略。努力将粤桂产业合作八步—怀集示范区上升到自治区层面加快推进。进一步加强桂东四市合作,争取自治区在重大产业布局和重点基础设施建设方面向桂东承接产业转移示范区倾斜。全面对接“桂粤湘赣”生态经济走廊战略构想,争取将贺州市纳入桂林国家旅游综合试验区建设,加快桂贺区域一体化发展。

　　进一步加强招商引资工作。加强与泛珠三角、台湾和东盟等重点区域的产业对接,抓好集群式产业转移。深化与中国铝业、中国有色、国电集团等央企名企合作,进一步提升项目履约率和开竣工率。深入开展招商引资大兑现工作,不断提高招商引资成效,确保实现投资规模500万元以上的招商引资项目到位资金310亿元以上,其中外资项目到位资金9700万美元以上。

　　八、提升生态文明水平,加快建设美丽贺州

　　全面推进节能减排。严格实行固定资产投资项目节能审查、环境影响评价制度。加强采矿区生态修复治理,开展粉体生产企业绿色生产活动,争取石材加工企业实现零排放。重点推进矿产、林产等行业企业的清洁生产,抓好火电、冶炼、水泥等行业的脱硫脱硝工程建设。加快淘汰落后产能,为发展新兴产业腾出环境容量。完善环境风险预警和应急处理机制,严防重大环境污染事故发生。

　　强化生态保护建设。实施主体功能区规划,划定生态保护红线。加强姑婆山、滑水冲、七冲、大桂山、西岭山五大自然保护区的生态环境保护,重点开展贺江、桂江、龟石水库等水环境建设,加大对饮用水水源地保护力度。广泛开展广西园林城市、园林县城和广西生态县(区)、生态乡镇(街道)、生态村创建活动。深入推进“国家森林城市”创建活动和“绿满贺州”造林工程,力争完成造林24万亩以上。

　　加快发展循环经济。深化与华润集团合作,继续吸纳关联项目进入贺州华润循环经济产业示范区,把园区建设和周边的休闲养生、旅游开发、新农村建设结合起来,建设成为综合发展、科学发展、城乡统筹的示范区。推进电力、建材、食品、电子、新材料等领域能源资源循环利用,加快发展太阳能、风能、生物质能等可再生能源。深入推广以沼气为纽带的生态循环农业模式。

　　九、注重坚持文化引领,努力创建文化先进城

　　提高城市文明水平。加快市文化中心、图书馆、博物馆、群众艺术馆建设,建设完善城区书报亭、乡镇综合文化站、村级公共服务中心、农家书屋等硬件设施。深化民族文化之乡、特色文化之乡、优秀村屯文艺队、小康文化示范户创建活动,培育良好的社会风尚。继续开展“国学进万家”活动,实施文化精品工程、“千村万户文艺惠民”工程,不断增强文化软实力。

　　培育壮大文化产业。加快建设桂台(贺州)客家文化旅游合作示范区,推进自治区级瑶族文化(富川、八步)生态保护区、临贺故城、姑婆山客家风情小镇、世界客家文化公园等项目建设。打造以客家文化、瑶族文化、茶文化、莲文化、石文化为重点的地方特色文化产品。

　　发展公益性文化事业。广泛开展群众性文化活动,打造“多彩贺州”大家演广场文化品牌,提高“村村通”的质量和水平。实施文化人才队伍“十百千”培养工程,加强市县乡村四级文化人才队伍建设。推进市县两级图书馆、博物馆、艺术馆和乡镇文化站免费开放工作。强化文化市场监管,坚决打击盗版、非法出版物,净化文化市场。

　　十、加强创新社会管理,切实保障改善民生

　　积极推进民生事业。加快实施第五轮创新计划和知识产权战略。深入实施中心城区教育项目建设大会战,确保8所新建、扩建中小学校按计划推进。继续巩固医改成果,有序开展公立医院综合改革试点工作,加快推进县乡村三级医疗卫生机构建设。促进群众体育和竞技体育、体育设施和体育产业协调发展。加快人口计生综合改革,促进人口长期均衡发展。加大170个贫困村整村推进扶贫开发力度,加快实施钟山县等革命老区扶贫攻坚三年帮扶行动。加快推进驻梧管理处大院危旧房改造。部署实施2013年为民办实事工程,加大社保、教育、医疗、安居等方面惠民力度。综合运用多种调控手段,建立健全价格监管长效机制,保持物价总体水平基本稳定。

　　提高社会管理水平。以创建“社会和谐稳定模范城”为契机,扎实推进“平安贺州”、“法治贺州”建设,建设具有贺州特点的社会管理体系,努力在矛盾纠纷排查调处、公共安全防控体系、基层基础建设、信息网络管理、流动人口和特殊人群管理、社会组织管理等方面实现新突破。加强国防动员、国防后备力量建设工作,推进双拥共建活动,进一步推进军民融合式发展。深入推进“六五”普法,提升公民法治意识。着力推进信访积案化解,完善乡镇、村、屯三级信访维稳工作网络。健全重大决策社会稳定风险评估机制,加强市、县、乡三级政府应急体系建设。完善食品药品安全监管体制机制,深入开展食品药品安全专项整治。强化公共安全体系和企业安全生产基础建设,严格落实各项安全生产责任制度。

　　扎实做好其他工作。充分发挥工会、共青团、妇联等人民团体和社会组织参与管理、服务的重要作用,认真落实党的民族、宗教和侨务政策,进一步做好统计、烟草、气象、水文、老龄、残联、档案、地方志、机构编制、行政服务、决策咨询、驻梧管理、住房公积金、检验检疫、库区移民、防震减灾等工作。

　　各位代表,完成繁重的工作任务,必须进一步强化政府自身建设,努力打造团结、高效、廉洁、开放政府。我们将全面贯彻落实市委决策,自觉接受人大及其常委会的法律监督和工作监督、政协民主监督、新闻舆论监督和社会公众监督,广泛听取各民主党派、工商联、无党派人士等社会各界意见。坚决落实中央关于改进工作作风、密切联系群众的八项规定,切实改进会风文风作风,对确定的事项,以雷厉风行的作风抓落实；对急难险重工作,以勇于担当的勇气抓落实；对重大项目和民生实事,以定责问效的手段抓落实。加大行政监察和审计监督力度,切实加强工程建设、土地出让、政府采购、产权交易等重点领域监管,严格控制“三公”经费,严格履行“一岗双责”和廉洁自律各项规定,严肃查处各类违法违纪案件。深入推进政务公开,突出把教育、医疗卫生、计划生育、供水、供电、环保、通信、公共交通等民生问题作为政府信息公开的重点,更加注重政民互动,不断增进相互沟通和理解。

　　各位代表!推动科学发展、实现增收目标、建设美丽贺州任务繁重、责任重大。让我们在自治区党委、政府和市委的领导下,紧密团结依靠全市各族人民,大力弘扬“重诚厚德、开放包容、自强不息”的贺州精神,开拓创新、艰苦奋斗、务求实效,以更加优异的成绩开启贺州发展新十年,力争与全国全区同步全面建成小康社会!
